# Correction: “First-Principles Investigation of Surface p*K*_a_ and the Behavior of Acids at Aqueous–Metal Interfaces”

**DOI:** 10.1021/acs.jpcc.5c02312

**Published:** 2025-04-11

**Authors:** Basil Raju Karimadom, Dan Meyerstein, Amir Mizrahi, Haya Kornweitz

The calculated surface p*K*_a_ (*p*K*_a_) values were originally underestimated (contained in Table 1, Table 2, Table 3, Table S4 and Figure 2), and the corrected values are given in Table 1 and the corrected Figure 2 below. The correction in values is due to the usage of the incorrect value of gas constant *R* in eq 10 for calculating *p*K*_a_. The recalculated values shifted toward lower values than the previous values, and it shows the strong acid behavior of weak acids on the metal–aqueous interface than those in the homogeneous solution. Hence, the corrections do not change the data conclusion in the original manuscript.

**Figure 2:**
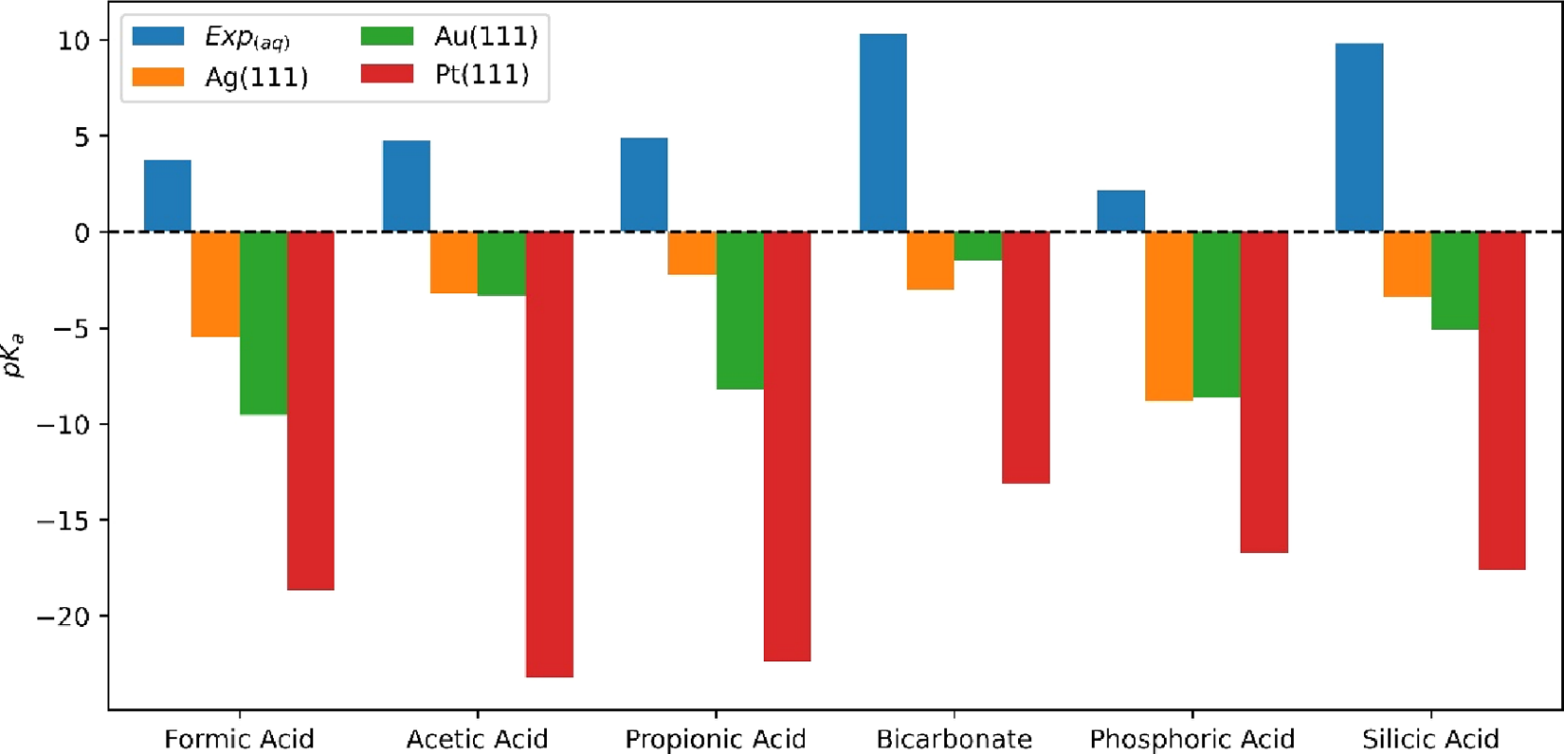
Corrected *p*K*_a_ of acids on M(111) surfaces.

**Table 1:** Surface p*K*_a_ Values (*p*K*_a_) of Various Acids (AH) on the M(111) Surface

**Surface**	**AH**	*p***K***_**a**_	*p***K***_**a2**_
**Ag(111)**	HCOOH	–5.46	
	CH_3_COOH	–3.18	
	CH_3_CH_2_COOH	–2.25	
	HCO_3_^–^	–3.03	
	PO(OH)_3_	–8.83	–0.87
	Si(OH)_4_	–3.39	–2.35
	H_3_O^+^	2.66	
**Au(111)**	HCOOH	–9.54	
	CH_3_COOH	–3.29	
	CH_3_CH_2_COOH	–8.21	
	HCO_3_^–^	–1.51	
	PO(OH)_3_	–8.62	–6.94
	Si(OH)_4_	–5.07	–4.79
	H_3_O^+^	a	
**Pt(111)**	HCOOH	–18.65	
	CH_3_COOH	–23.22	
	CH_3_CH_2_COOH	–22.37	
	HCO_3_^–^	–13.09^b^	
	PO(OH)_3_	–16.68	–18.29
	Si(OH)_4_	–17.61	–15.86
	H_3_O^+^	–4.31	

a The *p*K*_a_ cannot be calculated on the Au surface as H_3_O^+^ is not adsorbed on the Au surface.

b The *p*K*_a_ at 0.019 particle/Å coverage is −10.62 and at 0.038 particle/Å coverage is 8.68.

